# Sagittal Full-Spine vs. Sectional Cervical Lateral Radiographs: Are the Measurements of Cervical Alignment Interchangeable?

**DOI:** 10.3390/jcm13092502

**Published:** 2024-04-24

**Authors:** Jason W. Haas, Paul A. Oakley, Joseph W. Betz, Jason E. Miller, Jason O. Jaeger, Ibrahim M. Moustafa, Deed E. Harrison

**Affiliations:** 1 CBP NonProfit, Inc., Eagle, ID 83616, USA; drJasonhaas@gmail.com (J.W.H.); drjoebetz@gmail.com (J.W.B.); drmiller@posture-works.com (J.E.M.); drjaeger@cox.net (J.O.J.); 2 Kinesiology and Health Science, York University, Toronto, ON M3J 1P3, Canada; docoakley.icc@gmail.com; 3 Private Practice, Boise, ID 83709, USA; 4 Private Practice, Lakewood, CO 80226, USA; 5 Community Based Internship Program, Associate Faculty, Southern California University of Health Sciences, Whittier, CA 90604, USA; 6 Department of Physiotherapy, College of Health Sciences, University of Sharjah, Sharjah 27272, United Arab Emirates; 7 Neuromusculoskeletal Rehabilitation Research Group, RIMHS–Research Institute of Medical and Health Sciences, University of Sharjah, Sharjah 27272, United Arab Emirates

**Keywords:** cervical spine, neck pain, cervical lateral radiograph, whole-spine lateral radiograph, cervical lordosis, sagittal balance, radiography, X-ray

## Abstract

**(1) Background:** This study assessed the relationship between cervical spine parameters taken on standing full-spine lateral radiographic images compared to sectional lateral cervical radiographs. **(2) Methods:** Full-spine (FS) and sectional lateral cervical (LC) radiographs from four spine treatment facilities across the USA retrospectively provided data collected on 220 persons to assess the comparison of three sagittal cervical radiographic measurements between the two views. The measures included cervical lordosis using the absolute rotation angle from C2-C7, sagittal cervical translation of C2-C7, and atlas plane angle to horizontal. Linear correlation and R^2^ models were used for statistical comparison of the measures for the two views. **(3) Results:** The mean values of the three measurements were statistically different from each other: C2-C7 translation (FS = 19.84 ± 11.98 vs. LC = 21.18 ± 11.8), C2-C7 lordosis (FS = −15.3 ± 14.63 vs. LC = −18.32 ± 13.16), and atlas plane (FS = −19.99 ± 8.88 vs. LC = −22.56 ± 8.93), where all values were *p* < 0.001. Weak-to-moderate-to-strong correlations existed between the full-spine and sectional lateral cervical radiographic variables. The R^2^ values varied based on the measurement were R^2^ = 0.768 (*p* < 0.001) for sagittal cervical translation of C2-C7 (strong), R^2^ = 0.613 (*p* < 0.001) for the absolute rotation angle C2-C7 (moderate), and R^2^ = 0.406 (*p* < 0.001) for the atlas plane line (weak). Though a linear correlation was identified, there were consistent intra-person differences between the measurements on the full spine versus sectional lateral cervical radiographic views, where the full-spine view consistently underestimated the magnitude of the variables. **(4) Conclusion:** Key sagittal cervical radiographic measurements on the full spine versus sectional lateral cervical radiographic views show striking intra-person differences. The findings of this study confirm that full spine versus sectional lateral cervical radiographic views provide different biomechanical magnitudes of cervical sagittal alignment, and caution should be exercised by health care providers as these are not interchangeable. We recommend the LC view for measurement of cervical sagittal alignment variables.

## 1. Introduction

Valid and reliable assessment of the biomechanics and structural integrity of the spine and surrounding tissues is critical in the fields of surgery, advanced diagnostic imaging, and spine instrument manufacturing and for those rehabilitation specialists who use specific loading vectors via traction and other forms of force-based manipulation to address segmental and regional spine displacements [1,2,3,4,5,6,7]. Improper evaluation of underlying spinal biomechanics may lead to the development of cervical spine and spine disorders, which account for some of the highest global burden of disease and represent a significant cause of suffering globally [8]. Together, low-back pain and neck pain (NP) accounted for USD134.5 billion spent in 2017 in the USA, the highest of all the conditions studied [9]. Despite this significant financial and human suffering burden, NP research is significantly underfunded, and more research is needed to assess proper evaluation and treatment [10].

Comparison between the standing full-spine (FS) lateral radiograph and the dedicated lateral cervical (LC) radiograph has only been studied minimally in the medical literature. Those previous studies failed to conclude whether dedicated LC views are necessary to be obtained for a more accurate assessment of radiographic parameters needed for biomechanical assessment and treatment considerations. Lee et al. found that the standard hands-on-clavicles full-spine lateral radiographic position can make assessment of T1 slope and other parameters difficult to impossible [11]. Further, the hands-on-clavicles radiographic FS position has preliminarily been reported to alter the measurement amount of the cervical sagittal balance, patient-specific cervical lordosis, and altered head posture; each of these variations might alter the diagnosis and treatment for surgeons and rehabilitation procedures alike [11,12]. Park et al. found that the C2-C7 lordosis angle, C7-sternal angle, T1 slope, C2 offset translation distance, and C4 A-P diameter were all different when comparing the LC and FS views [12].

Despite the finding of differences comparing LC and FS views, these two prior studies did not make a conclusive argument for the superiority/necessity of one radiographic view compared to another [11,12]. This leaves health care providers with no clear path as to the clinical utility of the biomechanical analysis of sagittal cervical spine alignment from radiographs when data are obtained from lateral cervical versus lateral full-spine views. There is no general acceptance on the most valid method radiographic view of determining sagittal cervical spine alignment, nor are there published recommendations in the literature on the comparison of evaluation methods obtained from LC vs. FS procedures. For example, publications frequently inform surgeons of data implying they are from sectional LC views when they are referencing papers that used lateral full-spine views [13]. This frequent interchanging of data from LC vs. FS radiographs may have implications that are important to clinicians and, of course, clinical outcomes. This study seeks to fill gaps in the literature and help guide health care providers on the appropriateness of interpreting data obtained from LC versus FS radiographs in the clinical setting.

Finally, the sample size in the previous investigations were limited and were only collected from one facility, making comparisons across multiple spine centers limited [11,12]. The current investigation, therefore, presents a multi-center comparison of FS to LC in the same patient. We hypothesize that there will be significant variability in key sagittal cervical spine radiographic alignment variables between the FS and LC radiographic views. The primary aim of our investigation is to identify the most accurate radiographic view for the biomechanical assessment of key sagittal cervical spine alignment variables.

## 2. Materials and Methods

### 2.1. Patient Data Collection

We retrospectively reviewed patient demographic and radiographic data from four spine clinics in the USA. Informed consent, as per government regulations in the USA, was not required since this is a retrospective review of clinical records and is exempt from IRB approval under section 45 CFR 46.101(b)(4). See www.hhs.gov/ohrp/regulations-and-policy/decision-charts-pre-2018/index.html#c5 (accessed on 17 March 2024). Each patient received a standard physical assessment including radiographic examination. Over the years 2012–2014, patients were retrospectively but consecutively collected based on the criteria that each patient had both a full-spine lateral weight-bearing radiograph (FS) and a dedicated sectional lateral cervical spine radiograph (LC) where all cervical structures were clearly visible; patients were 18 years of age or older. As part of their examination, general informed consent and authorizations were completed by each patient as part of their patient intake forms in each facility.

Patient data collected included a numerical pain rating scale (NPRS) [14], height, weight, gender, and radiographic characteristics reported for this study. Patients with missing demographic data or with radiographs that were of inferior diagnostic quality rendering anatomical points unidentifiable or with anatomical points not within the margins of the films were excluded. Additionally, patients with significant pathologies or spinal anomalies, as discussed below, were also excluded. The four spine treatment centers retrieved a total of 220 consecutive patients meeting these inclusion and exclusion criteria. The facilities which provided clinical data included:

Facility 1—Las Vegas, NV, USA. (n = 50).

Facility 2—Windsor, CO, USA. (n = 30).

Facility 3—San Francisco, CA, USA. (n = 40).

Facility 4—Eagle, ID, USA. (n = 100).

### 2.2. Radiographic Evaluation Procedure

The spine treatment centers provided FS and LC radiographs and patient data for this study. Patient data were collected and provided by licensed physicians with legal authorization via federal Medicare laws of the USA to diagnose and recommend treatment for spine disorders, including the use of diagnostic spinal X-rays. All licensed physicians at the four locations had a minimum of ten years of experience taking spine radiographs in a clinical setting daily. Patient instruction and positioning were standardized between the four facilities with previous in-person training at the center located in Eagle, Idaho. The radiographic technique was standard best practice for both LC and FS [15].

LC was performed in a neutral posture with the patient standing with the shoulder gently touching the bucky (not leaning), the arms resting comfortably at the sides, and the central ray near the central cervical spine. The patient was not instructed to perform any re-positioning techniques beyond their normal position. The assessment was performed in the patient’s “neutral” position, meaning the patient was instructed to comfortably nod their head up and down, finding the neutral self-balanced position, where they would then open their eyes and maintain an eye-level sightline. The FS radiograph was performed in two manners, based on the individual needs: (1) if the patient was noticed to sway in the sagittal plane in the neutral upright position with hands resting on their ipsilateral clavicles, the patient was instructed to gently rest their hands on a bar at waist level with elbows slightly bent without reaching forwards, and (2) patients who did not have noticeable sagittal sway were instructed to gently place the fingers on their ipsilateral clavicles with a limited arm raise. In both positions, the patient’s shoulder gently touched the bucky (not leaning), and the patient was instructed to not alter any neutral position and was merely instructed to look straight ahead and not move from that position. No separation of these two FS positioning procedures was tracked for each patient such that it is unknown how many of the 220 patients received each one.

LC radiographs were included if the landmarks were clear; that is, if all pertinent points from the upper cervical spine to the sternum and T1 could be clearly visualized. Further, LC radiographs were included if they were void of significant pathologies/anomalies (i.e., space occupying lesions, malignancies, congenital malformations, anatomical anomalies, etc.). FS radiographs were included if all the points necessary for evaluation, including the entire cervical spine to the lumbo-pelvic spine, could be clearly visualized. All vertebral body corners were required to be clearly visualized for mensuration. Exclusion criteria for the FS were the same as for the LC. As the visualization of T1–T4 is frequently the most difficult on the FS and T1 on the LC, the inability to visualize these segments were the most frequently determined reasons for exclusion. Approximately 15% of the films were excluded for poor visualization reasons.

**Figure 1:**
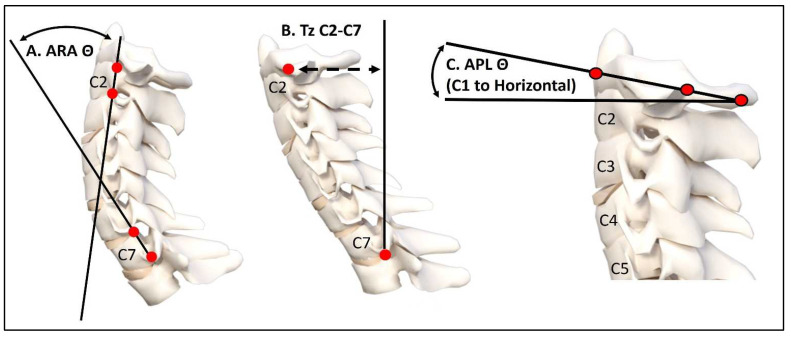
Sagittal cervical spine radiographic variables measured with the PostureRay^®^ software. In (**A**), the ARA C2-C7 measurement of cervical lordosis is shown. In (**B**), the translation distance of C2 relative to C7 is shown. In (**C**), the atlas plane line (APL) to horizontal is shown. These measurements have excellent examiner reliability [16,17].

**Figure 2:**
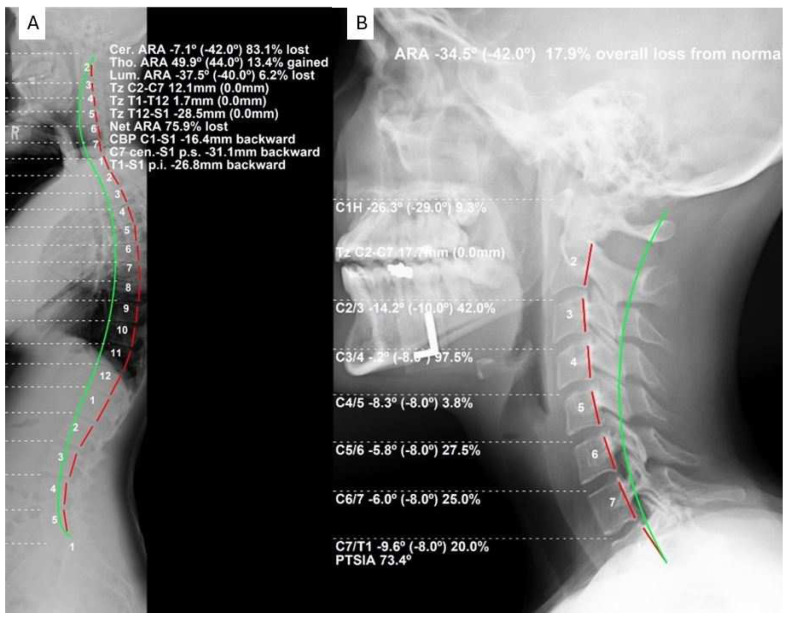
(**A**) shows a sample patient full-spine lateral radiograph (FS) analyzed using the posterior tangent method with PostureRay^®^ software. The ARA C2-C7 measurement of cervical lordosis is −7.1°, which is significantly less than the cervical lateral radiograph of the same patient in (**B**). (**B**) shows a sample lateral cervical radiograph (LC) of the same patient as in (**A**) above, showing a much less distorted image with greater accuracy. The ARA C2-C7 is a deeper lordosis measuring −34.6°, nearly five-fold greater than the FS measurement.

The radiographic mensuration assessment was completed using PostureRay^®^ (PostureCo Inc.^®^, Trinity, Fl, USA). Assessments were made for absolute rotation angle (ARA) from C2-C7 on the LC and FS using the Harrison posterior tangent method [15,16]. The atlas plane line of C1 to horizontal (APL) was measured on both views. Anterior-posterior head translation (+/−TzH) was measured as the linear distance between a vertical axis line arising from the posterior inferior vertebral body corner of C7 and the posterior superior vertebral body corner of C2. Figure 1 depicts the three measurements used for the assessment of the key lateral cervical radiographic alignment data. Figure 2A represents a sample full-spine lateral radiograph, and Figure 2B shows the sectional lateral cervical radiograph of the same individual as in Figure 2 for a visual comparison of obtained values for these measurements.

### 2.3. Statistical Analytical Methods

Patient descriptive data and radiographic variables are reported as means and standard deviations. Data for each of the three variables on each of the two views is presented with visual context using boxplots and histograms to visually assess their distribution and fit or no-fit to a normal distribution. Patient data were initially imported into Microsoft Excel (2018 Microsoft Excel. Retrieved from www.office.microsoft.com/excel accessed on 2 January 2024), and statistical analysis was performed with SPSS version 29. To determine the normality of the collected numerical variables, the Kolmogorov–Smirnov and the Shapiro–Wilk tests were used. Differences between the means were calculated via repeated measures *t*-tests for normally distributed variables or else by the related-samples Wilcoxon signed-rank test for variables not normally distributed. Cohen’s d effect sizes were calculated to examine the differences in the means and standard deviations of the three variables on the two radiographic views, where d ≈ 0.2 indicates small effect, d ≈ 0.5 indicates medium effect, and d ≈ 0.8 indicates a large effect [18].

Due to the known limitations of Cohen’s d effect size [18], in order to accurately assess the statistical relationship between the paired radiographical variables (LC vs. FS), when the data were normally distributed, the Pearson r correlation coefficient was used; otherwise, for not normally distributed data, the Spearman ranked correlation coefficient (r) was used. The level of significance was set at 0.05, and any correlation was considered statistically significant when the *p*-value < 0.05. Finally, the R^2^ linear regression model was used to compare two radiographic views for each variable to each other to determine the actual statistical fit in the intra-person data and percentage variation between the two radiographic views for each of the three measured variables. Regression equations for the values of FS were reported to predict the corresponding value for LC.

## 3. Results

### 3.1. Patient Demographics

**Figure 3:**
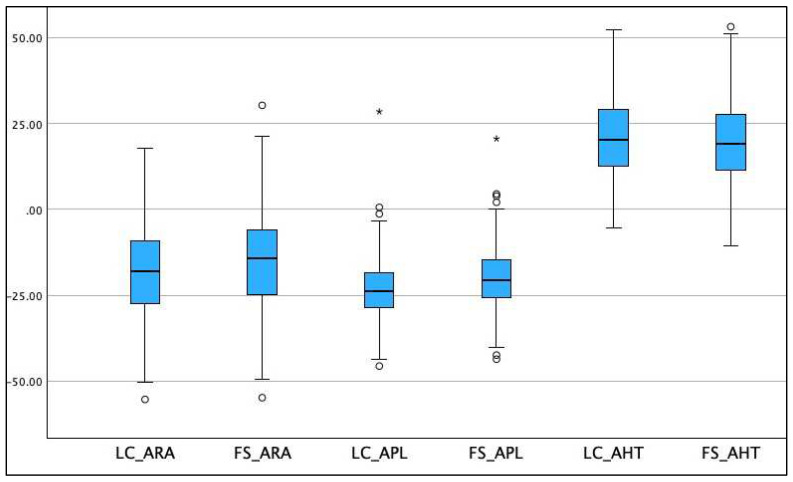
Box plots for the distribution of the absolute rotation angle (ARA C2-C7), atlas plane line (APL), and anterior head translation (AHT C2-C7) on the sectional lateral cervical (LC) radiograph compared to the full-spine (FS) lateral radiograph. The blue-shaded box represents the second and third quartiles. The lower and upper whiskers go from the minimum to the lower quartile (the inferior margin of the box) and then from the upper quartile (the top of the box) to the maximum. Units are degrees (°) for angles (ARA; APL) and millimeters (mm) for distances (AHT).

The four clinical data collection centers produced 220 patients for analysis. Table 1 shows the descriptive demographic and clinical variables. Gender characteristics indicated that 36.9% were male and 63.1% female. The means and standard deviations are shown for the three radiograph measurement variables assessed on the two radiographic views: the full-spine lateral (FS) and sectional lateral cervical (LC). Box plots for the distribution of the three radiographic variables (AHT, ARA, and APL) are shown for the two radiographic views (FS and LC) as a side-by-side visual comparison of the distributions of these radiographic data; see Figure 3.

Differences between the means of the LC and FS views for the ARA and AHT were calculated using repeated measures *t*-tests, as these variables were normally distributed. The mean ARA for the LC was statistically greater than the mean for the FS, t(219) = 4.856, *p* < 0.001, and the mean AHT for the LC was statistically greater than the mean for the FS, t(219) = 3.385, *p* < 0.001 (Table 1). The difference between means of the LC vs. FS for the APL was calculated using the related-samples Wilcoxon signed-rank test and was statistically significantly greater for the LC view (*p* < 0.001) (Table 1).

### 3.2. Normality of Distribution of the Radiographic Data

**Figure 4:**
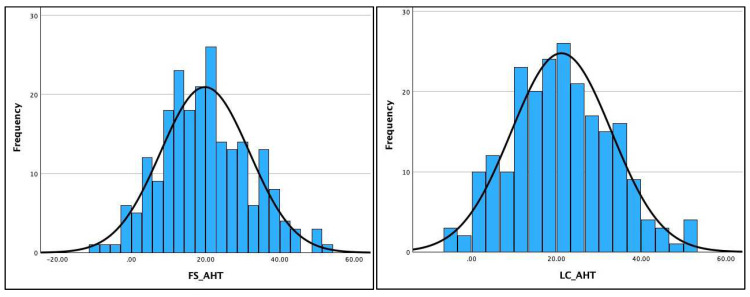
Histograms for anterior head translation (AHT) C2-C7 on the full-spine (FS) lateral versus the lateral cervical (LC) radiographs. Units are in millimeters.

**Figure 5:**
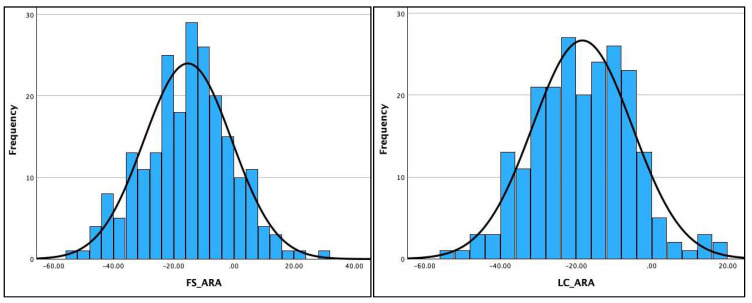
Histograms for absolute rotation angle (ARA) C2-C7 for cervical lordosis on the full-spine (FS) lateral versus the lateral cervical (LC) radiographs. Units are in degrees.

**Figure 6:**
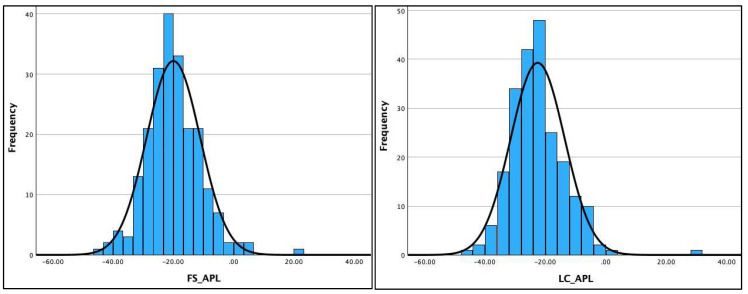
Histograms for atlas plane line (APL) to horizontal for upper-cervical lordosis assessment on the full-spine (FS) lateral versus the lateral cervical (LC) radiographs. Units are in degrees.

Table 2 shows the Kolmogorov–Smirnov and the Shapiro–Wilk test results for assessing the normality of distribution of the three radiographic variables (AHT C2-C7, ARA C2-C7, and APL) on the two different radiographic views. The data were normally distributed for four out of six variables (ARA C2-C7 and AHT C2-C7) on both radiographic views and is clearly visualized in the histograms for the AHT and ARA variables in Figure 4 and Figure 5. However, the data violated the terms of normality for the APL on both radiographic views, shown in the histogram for the APL in Figure 6 and detailed in Table 2. As a result, the Pearson’s r correlation was determined to assess the statistical relationship between these variables on the two views: ARA C2-C7, r = 0.785 (*p* < 0.001) and AHT C2-C7, r = 0.877 (*p* < 0.001). The data for the APL violated the test for normal distribution; thus, the Spearman ranked correlation (r) was determined to assess the statistical relationship between the two radiographic views for APL: APL, r = 0.703 (*p* < 0.001).

### 3.3. Regression Analysis with R^2^

**Figure 7:**
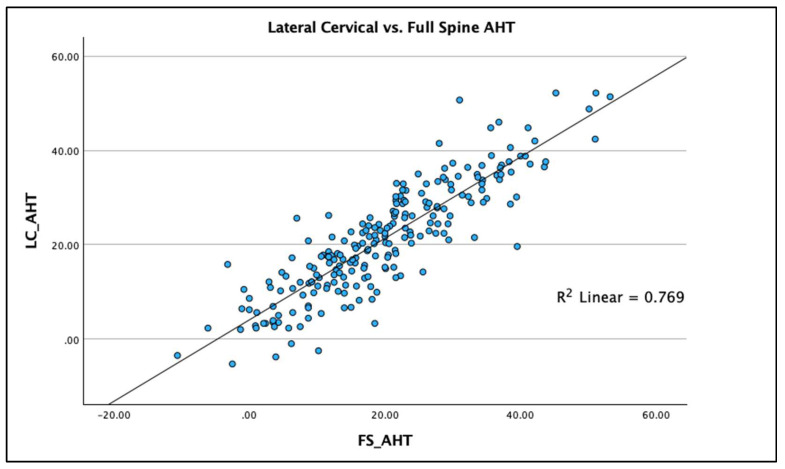
Linear regression (with R^2^ value) scatter plot of anterior head translation (AHT) from C2-C7 on the sectional lateral cervical (LC) radiograph compared to the full-spine (FS) radiograph. Units are in millimeters.

**Figure 8:**
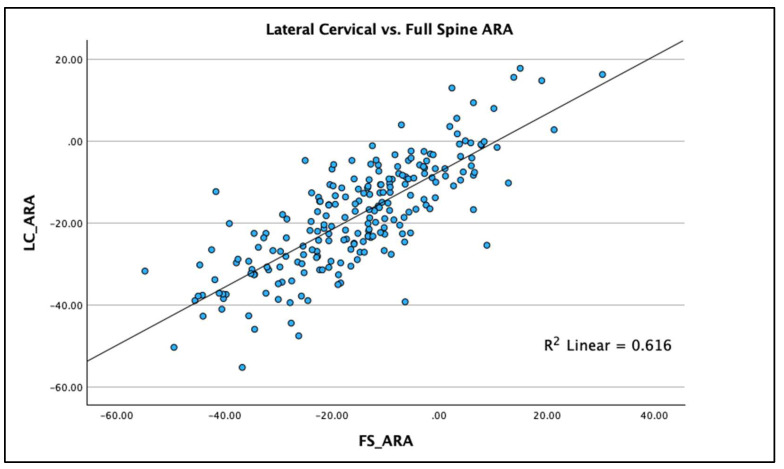
Linear regression (with R^2^ value) scatterplot of absolute rotation angle (ARA) from C2-C7 on the sectional lateral cervical (LC) radiograph compared to the full-spine (FS) radiograph. Units are in degrees.

**Figure 9:**
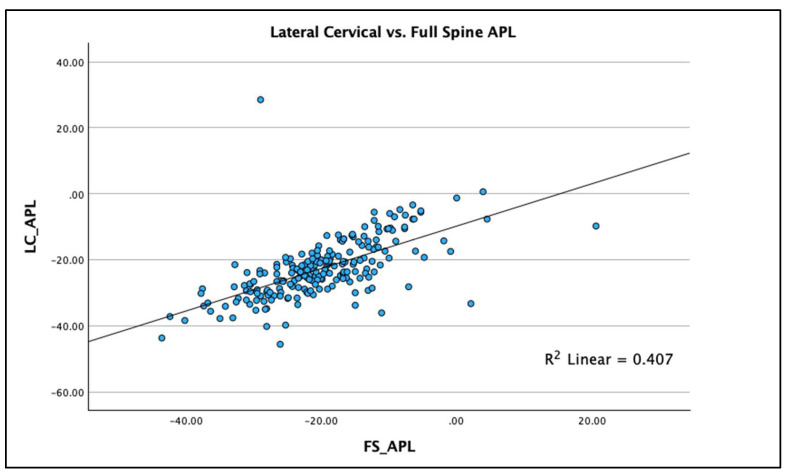
Linear regression (with R^2^ value) scatterplot of the atlas plane line (APL) to horizontal on the sectional lateral cervical (LC) radiograph compared to the full-spine (FS) radiograph. Units are in degrees.

R^2^ linear regression analysis was used to compare the two radiographic views and the three measured variables on each view to determine the statistical fit and percentage variation between the two radiographic views for each of the three measured variables. Results are graphed using scatterplots with the R^2^ regression line representing the ordinary least squares method for the pairs of views for each of the three spine parameters. The regression analysis allows evaluation of accuracy (“goodness of fit”) of the comparisons between views. Scatterplots with linear regression lines clearly demonstrate the analysis findings with clear clustering of data points along the regression line with the greatest R^2^ value being for AHT C2-7 (R^2^ = 0.769 *p* < 0.001), followed by ARA C2-C7 (R^2^ = 0.616; *p* < 0.001) and APL (R^2^ = 0.407; *p* < 0.001), Figure 7, Figure 8 and Figure 9 depict this analysis.

Since the gold standard assessment of the cervical spine is a dedicated LC view, the regression equations assigning the FS variables as independent variable predictors and the LC variables as the dependent variables are as follows:

ARA: y = −7.52 + 0.71*x

APL: y = −9.82 + 0.64*x

AHT: y = 4.04 + 0.86*x

## 4. Discussion and Clinical Interpretation

The current investigation presents a multi-center comparison of full-spine (FS) lateral radiographs to lateral cervical (LC) sectional radiographs in the same patient. We hypothesized that there would be significant variability in key sagittal cervical spine radiographic alignment variables between the FS and LC radiographic views. In 220 patients, across four spine centers, we identified that there were, indeed, statistically significant differences in three key sagittal cervical spine measurement variables between the LC and FS radiographic views. The FS radiograph was found to underestimate the amount of anterior head translation (AHT C2-C7), underestimate the amount of cervical lordosis (ARA C2-C7), and underestimate the amount of extension of the upper cervical spine (APL). Our investigation adds to the literature determining the most appropriate and clinically relevant radiographic view to use for the most accurate cervical spine parameters; namely, we find that a sectional LC radiograph is the most important view to determine sagittal cervical spine alignment, and caution should be used when interpreting the same variables on the FS radiograph. Having knowledge of this information can assist treatment recommendations for neck pain and other spine conditions, and our findings add validity for the necessity for both the LC radiograph and the FS radiograph to accurately assess spine parameters prior to clinical interventions; a clear presentation of our findings and comparison to the existing literature follows [11,12,19,20,21].

### 4.1. Previous Investigations with Comparison to Current

Our literature search identified five previous investigations from 2015 to 2021 that have looked at alignment variations of sagittal cervical spine radiographic measures between the FS and LC views [11,12,19,20,21]. In 2015, Park et al. [19] presented the first report in the literature using a prospective database of 101 asymptomatic adult volunteers. They identified statistically significant differences between the FS (hands-on ipsilateral clavicular fossae position) and LC radiographs, where the LC showed greater C2-C7 ARA and Cobb angles, greater translation of C2-C7 on the LC, greater T1 slope on the LC, and slightly more head flexion on the FS view [18]. In 2016, Park and colleagues [12] retrospectively reviewed 59 adults with chronic neck pain for their comparison of cervical measurements on the LC vs. FS (hands-on ipsilateral clavicular fossae position) radiographs. They found statistically significant differences in the C2-C7 Cobb angle, the C7-sternal angle, and the C2-C7 translation distance but claimed that the differences between these variables and views were within the inter-examiner error margin for assessing the films [12]. In 2019, Morimoto et al. [20] presented the first comparison of seated sectional LC views compared to FS standing radiographs (hands-on ipsilateral clavicular fossae position) in 50 patients with a variety of pre-surgical spine deformities, including fractures. They identified that McGregor’s head flexion angle was more flexed on the FS view and the T1 slope showed more extension (smaller angle) on the FS view. No significant differences in C0-C2 and C2-C7 Cobb angles were identified; however, a different statistically significant correlation (Pearson’s r) was identified from C2-C7 and to the T1 slope between the two views, indicating a rather different fit of the cervical lordosis to the T1 vertebral slope [20].

In 2019, Lee and colleagues [21] presented a retrospective analysis of 71 adult participants with mild neck and back pain, where all spine deformity was excluded. Problematically, they used two different methods for FS view positioning, the hands on the ipsilateral clavicles and the arms extended straight out at 90° relative to the horizontal, and no mention of accounting for these two positioning was reported. However, Lee et al. [21] did assess the difference of subgroups of their population with and without more than a 5° downward head tilt between the FS and LC views. They identified significant differences where the FS view showed increased C2 slope, increased C0 flexion, increased C0-C2 flexion, a flatter C0-C7 Cobb angle on the FS, hypo-lordosis of C2-C7 Cobb angle on the FS, and less C2-C7 translation on the FS view [21]. Lastly, in 2021, Lee et al. [11] revisited the topic of sectional LC vs. FS radiographs in a retrospective review of 60 patients suffering from neck pain, radiculopathy, and myelopathy. They identified no differences in the T1 and C7 vertebral body tilt (slope) to horizontal and no difference in the upper cervical lordosis from C0-C2. However, statistically significant differences were found with smaller C2-C7 Cobb angle lordosis on the FS view and more posterior translation (less anterior) of the C2-C7 distance on the FS view [11].

### 4.2. Significance of Current Investigation

Our current investigation’s results, based on the largest sample size available to date, are in general agreement with the previous literature, with a few exceptions [11,12,19,20,21]; however, we advance the understanding of the intra-person errors in measurements of cervical alignment on the sectional LC vs. the FS views due to our reporting of the R^2^ statistical analysis. We used a standard positioning of the lateral cervical radiographic view and the hands in the clavicular fossae, of which both views have been found to have good to excellent examiner reliability for positioning [22,23] and measurement [17,19]. Our current investigation identified that the FS lateral radiograph underestimates the amount of upper cervical lordosis, underestimates overall cervical lordosis, and underestimates the amount of anterior translation of C2-C7, and these findings are consistent with the above presented data [11,12,19,21] with minor exceptions, mostly from Morimoto et al. [20], likely due to the type of deformity population used (cervical myelopathy) and the decreased accuracy in identification of anatomical points in subjects with increased prevalence of bony degeneration of vertebral bodies.

Importantly, the previous literature only assessed the significance of differences using the mean and standard deviation of the measurement on the FS vs. the LC radiographs [11,12,19,20,21]. Similarly, we report these means and standard deviations along with their respective effect sizes in Table 1. Problematically, many authors ignore the fact that effect sizes are rather abstract statistics not immune from bias due to sampling and quality; effect sizes cannot differentiate between variable relationships of a similar magnitude that may have a different practical significance and relative intra-person difference [18]. Thus, the rather simplistic assessment of means and SDs does not elucidate the true intra-person difference of measurements for the cervical spine between the two radiographic views. For this reason, the current investigation used linear regression analysis with the R^2^ model value, also called coefficient of determination, which represents the proportion of variance (in %) in the measurements obtained for the dependent variable (LC) that can be explained by the independent variable (FS). Generally, a rule of thumb for interpreting the relative strength of a relationship based on its R^2^ value is the following four significances: (1) no or a very weak effect size is R^2^ < 0.3; (2) a weak effect size is 0.3 < R^2^ < 0.5; (3) a moderate effect size is 0.5 < R^2^ < 0.7; and (4) a strong effect size is given by R^2^ > 0.7 [24].

Using the above interpretation of the significance of the R^2^ value [24], we identified the following: (a) the R^2^ value for AHT C2-7 (R^2^ = 0.769 *p* < 0.001) is strong for the two radiographic views; (b) the R^2^ value for ARA C2-C7 (R^2^ = 0.616; *p* < 0.001) is moderate for the two radiographic views; and (c) the R^2^ value for APL (R^2^ = 0.407; *p* < 0.001) is weak for the two radiographic views. Our findings specifically indicate that anterior-posterior cervical translation measured with the C2-C7 plumbline (originally presented by Harrison et al. [25] and modified by others [11,12,19,20,21]) is suitable to be measured on both the FS and LC radiographic views, though some intra-person differences do exist that are significant (see Figure 7). However, considerable caution is advised in attempting to use the FS radiograph to measure the variables of cervical lordosis (ARA C2-C7 or Cobb C2-C7) and the upper cervical alignment (APL), as considerable intra-person differences exist between these measures and those obtained on the sectional LC radiograph; see Figure 8 and Figure 9 for an understanding of this. As a clear example of the large intra-person difference in cervical lordosis that can occur between the FS and LC films, Figure 2A,B is provided, where there is a five-fold increase in lordosis on the LC radiograph. It might be thought that the mild head flexion difference noted on the FS of the person in Figure 2 explains the reduction in overall cervical lordosis; however, this has been clearly refuted where mild head flexion does not change the magnitude of the cervical lordosis to this extent [19,26]. We argue that the FS radiograph alters the cervical alignment due to several variables that need further investigation, including projection distortion and magnification, shift of the whole-spine sagittal balance due to movement of the upper extremities out of the coronal plane, and a muscular effect from the shoulder and thoracic muscles interacting and connecting with the cervical spine musculature.

### 4.3. Clinical Implications

Radiography is critical in the evaluation, assessment of referral, and determination of proper treatment interventions for spine conditions [27,28]. Standing, plain film, X-ray spine radiography has been used for nearly a century to assess spine pain and recommend treatment [28,29]. The cause of neck and spine pain has been studied extensively, and there are numerous postulations as to the cause and concomitant disorders, such as genetic [30], morphologic [31,32], traumatic [33], and postural [34]. Recently, metabolic [35], nutritional [36], and psychosocial contributions to spine pain have also been explored [37]. The bio-psycho-social model is being studied more frequently; however, these and other mental health assessments must also consider the biomechanical contributions, as these have significantly more historical research and data and need to be evaluated primarily and simultaneously with any psycho-social and mental health models [38]. Similarly, radiography is still the objective criterion standard assessment for spine conditions [11,12,19,20,21,27,28,29,38], which, coupled with the outcome measures above, gives increased clinical certainty for proposed conservative and invasive procedures.

The current comparison study found that the differences between the FS and LC radiograph are significant enough to alter conservative and invasive clinical considerations. Angulation of cervical spine lordosis has been considered for many decades in spinal surgical procedures. Theoretically, failure to account for the measured differences between LC and FS could lead to improper surgical strategies aimed at improving cervical spine alignment (either secondarily or primarily), with the potential for devastating consequences [39,40,41,42,43,44,45]. Failed back surgery syndrome (FBSS) and persistent spine pain syndrome (PSPS) both are a result of surgical interventions that have significant consequences for patient and community and could potentially be avoided by better diagnostics [44,45,46]. In addition to an upright full-spine radiograph [44,45], we argue, based on the results of previous investigations [11,12,19,20,21] and the results of the current investigation, that imaging should now always include a sectional LC evaluation prior to interventions indirectly assumed to influence or directly aimed at improving cervical spine deformity alignment; this should also be the case for investigations on normative cervical spine alignment data [47,48,49]. The fact that publications assessing cervical spine alignment variables have used FS radiography is a considerable area for concern [50,51,52]. Spine alignment variables for the cervical spine need to be based on the sectional LC and not the FS radiograph. A final point on clinical insights for conservative rehabilitation specialists is that recent rehabilitation techniques consider that the exact alteration in sagittal cervical alignment (segmental, total curve shape, head translation magnitude, T1 slope, etc.) is a necessary assessment to determine the proper vector of extension traction forces applied to correct/restore proper cervical alignment [9,10,53,54,55,56].

### 4.4. Risk–Benefits of a LC and FS Radiograph

Prior investigations have evaluated the use and safety of plain film radiography in the diagnosis and treatment of spine conditions, based on the assumption that the use of X-ray radiation to produce an image of the patient would increase the incidence of diseases such as cancer [57,58,59]. The results from this study, however, show differences between the cervical spine parameters measured from LC versus FS radiographic views. The recommendation for obtaining an ‘extra’ dedicated lateral cervical view, beyond the standard full-spine lateral view should be obvious. An important consideration is whether it is worth the ‘risk’ considering the extra radiation exposure. Our answer is yes. First, though radiation exposure does cause damage via free radical production, it must be acknowledged that this is only about one one-millionth of the damage caused by normal metabolic processes occurring daily [60]. The largest exposure to oxidative damage occurs as a result of breathing air [61]. Second, the basis of radiation risk assessment is flawed by reliance on the linear no-threshold model, which is not scientific [62,63]; incredibly, it is anti-evolutionary [64] and simply invalid [65,66]. Third, the LNT hypothesis has recently been documented to be shrouded in controversy, as the basis relies on the Muller experiments that have been shown to be flawed [67,68]. Last, the benefit–risk ratio associated with an ‘extra’ radiograph to ensure precision in delivering contemporary spine care is always favorable to taking an extra image [60], not only for the added precision in making important clinical decisions, but also because there are no risks associated with modern medical radiography [69]. Judicious and proper use of X-ray radiation to obtain images of the full and sectional cervical spine and posture provides clinicians a tool to track the progress and/or degeneration of their patient’s spine and to assess the success or failure of their interventions, whether conservative or surgical [9,10,47,48,49,53,54,55,56].

### 4.5. Limitations

The limitations of this study are the narrow outcome measures of only three variables of cervical spine alignment for FS and LC radiographic comparison. Multiple parameters of comparison could be included in future projects, including T1 sagittal tilt, the thoracic inlet angle for morphological considerations, and segmental angles that could explain some of the differences between the FS and LC radiograph. Further, the positioning of the FS radiograph was performed in two manners (hands on the ipsilateral clavicular fossae and hands gently resting on a bar at hip height) based on the individual patient’s needs related to body sway. Both of these FS radiograph positioning procedures are reliable and represent a standard positioning in the sagittal plane [11,19,20,70,71]. No separation of these two FS positioning procedures was tracked for each patient, such that it is unknown how many of the 220 patients received each one. It is also unknown if these two methods of patient positioning result in clinically significant differences in radiographic biomechanical evaluation. Future studies will need to be performed to answer these questions. Finally, it might be thought the differences between the two X-ray views is merely due to the sizes of the different images. However, only distances would be altered by increased size due to project and angles would not, and both our LC and FS radiographs were taken at the standard tube to grid-cabinet distance of 72 inches so we doubt this was a significant reason for our findings.

## 5. Conclusions

This multi-center comparison study of upright lateral cervical analysis versus sagittal full-spine radiographs with specific analysis of the cervical spine is important to both conservative and surgical practitioners interested in the origination, evaluation, and treatment of spine disorders using radiography. We identified that the total cervical lordosis (ARA C2-C7) and upper cervical lordosis (APL) are strikingly different on the sectional LC vs. the FS radiograph when considering intra-person differences using the R^2^ analysis (only weak to moderate correlations were found). In contrast, we identified that anterior head translation (C2-C7 plumbline) showed strong correlation between the two radiographic views; thus, this can be considered relatively interchangeable between the LC and FS analysis. When considering any cervical spine conditions, it is crucial to not only evaluate the FS for global spine abnormalities and misalignments but also the LC sectional view to determine specific local spine biomechanics. Our findings suggest that spine alignment variables for the cervical spine need to be based on the sectional LC and not the FS radiograph. Data from one type of radiograph should not be compared with data from the other. Also, clinicians should only use data from the literature that is obtained as performed for the same type of radiograph (LC vs. FS). The interpretation of these comparative data demonstrates that the differences between measurements obtained from the LC and FS radiographs are significant enough to necessitate that the practitioner perform both assessments and compare them to determine the most appropriate clinical and diagnostic determination of the best treatment protocol. Failure to do so could be clinically detrimental in conservative care which uses traction vectors to improve spine alignment and could be especially detrimental to surgeons making pre-treatment decisions of cervical spine alignment based solely on the FS radiograph.

## Figures and Tables

**Table 1:** Descriptive data for the demographic variables are presented for 220 patients. The values are presented as mean and standard deviation (SD) for all variables except gender (%).

Variable	Mean ± SD			
Age (years)	43 ± 12.5			
Weight (kg)	74.9 ± 16.5			
Height (cm)	169.77 ± 10.76			
Neck pain intensity (NRS)	3.29 ± 2.08			
**Gender (%)**				
Male	36.9			
Female	63.1			
**Radiographic Variables**	**Full Spine**	**Lateral Cervical**	***p*-value**	**Cohen’s d ^c^**
AHT Tz C2-C7 (mm)	19.84 ± 11.98	21.18 ± 11.8	<0.001 ^a^	0.228 (0.094, 0.362)
ARA C2-C7 (°)	−15.3 ± 14.63	−18.32 ± 13.16	<0.001 ^a^	−0.327 (−0.463, −0.191)
Atlas Plane Line (APL) (°)	−19.99 ± 8.88	−22.56 ± 8.93	<0.001 ^b^	−0.352 (−0.489, −0.214)

Note: AHT: anterior head translation; ARA: absolute rotation angle; NRS: numerical rating scale 0–10. Full spine: full-spine lateral radiograph. Lateral cervical: sectional lateral cervical radiograph. ^a^: Repeated measures *t*-test. ^b^: Related-samples Wilcoxon signed-rank test. ^c^: Cohen’s d (95% confidence interval).

**Table 2:** The Kolmogorov–Smirnov and the Shapiro–Wilk test results for assessing normality of distribution of the three radiographic variables (AHT C2-C7, ARA C2-C7, and APL) on the two different radiographic views (the full-spine lateral (FS) and the sectional lateral cervical (LC)).

View and Variable	Kolmogorov–Smirnov ^a^			Shapiro–Wilk		
	Statistic	df	Sig.	Statistic	df	Sig.
**LC_ARA**	0.034	215	0.200 *	0.994	215	0.568
**FS_ARA**	0.045	215	0.200 *	0.996	215	0.850
**LC_APL**	0.084	215	<0.001	0.949	215	<0.001
**FS_APL**	0.052	215	0.200 *	0.980	215	0.004
**LC_AHT**	0.041	215	0.200 *	0.992	215	0.253
**FS_AHT**	0.056	215	0.200 *	0.992	215	0.277

* This is a lower bound of the true significance. ^a^. Lilliefors for significant correlation. Abbreviations: LC = lateral cervical sectional film, FS = full-spine lateral film, ARA = absolute rotation angle (C2-C7), APL = atlas plane line angle, AHT = anterior head translation.

## Data Availability

Additional pertinent data are available upon request.
